# A preventative lifestyle intervention for older adults (lifestyle matters): a randomised controlled trial

**DOI:** 10.1093/ageing/afx021

**Published:** 2017-02-25

**Authors:** Gail Mountain, Gill Windle, Daniel Hind, Stephen Walters, Anju Keertharuth, Robin Chatters, Kirsty Sprange, Claire Craig, Sarah Cook, Ellen Lee, Tim Chater, R. Woods, Louise Newbould, Lauren Powell, Katy Shortland, Jennifer Roberts

**Affiliations:** 1 School of Health and Related Research, University of Sheffield, Regent Court 30 Regent Street, Sheffield S1 4DA, UK; 2 Dementia Services Development Centre, University of Bangor, Wales, Bangor, UK; 3 School of Medicine, University of Nottingham, Nottingham, Nottinghamshire, UK; 4 Centre for Health and Social Care Research, Sheffield Hallam University, Sheffield, South Yorkshire, UK; 5 Sheffield NIHR Clinical Research Facility, Sheffield Teaching Hospitals NHS Foundation Trust, Sheffield, UK

**Keywords:** Older people, occupational health, randomised controlled trial, mental health

## Abstract

**Objectives:**

to test whether an occupation-based lifestyle intervention can sustain and improve the mental well-being of adults aged 65 years or over compared to usual care, using an individually randomised controlled trial.

**Participants:**

288 independently living adults aged 65 years or over, with normal cognition, were recruited from two UK sites between December 2011 and November 2015.

**Interventions:**

lifestyle Matters is a National Institute for Health and Care Excellence recommended multi-component preventive intervention designed to improve the mental well-being of community living older people at risk of decline. It involves weekly group sessions over 4 months and one to one sessions.

**Main outcome measures:**

the primary outcome was mental well-being at 6 months (mental health (MH) dimension of the SF-36). Secondary outcomes included physical health dimensions of the SF-36, extent of depression (PHQ-9), quality of life (EQ-5D) and loneliness (de Jong Gierveld Loneliness Scale), assessed at 6 and 24 months.

**Results:**

data on 262 (intervention = 136; usual care = 126) participants were analysed using intention to treat analysis. Mean SF-36 MH scores at 6 months differed by 2.3 points (95 CI: −1.3 to 5.9; *P* = 0.209) after adjustments.

**Conclusions:**

analysis shows little evidence of clinical or cost-effectiveness in the recruited population with analysis of the primary outcome revealing that the study participants were mentally well at baseline. The results pose questions regarding how preventive interventions to promote well-being in older adults can be effectively targeted in the absence of proactive mechanisms to identify those who at risk of decline.

**Trial Registration:**

ISRCTN67209155.

## Introduction

Current national guidance advocates the implementation of health promoting interventions for older people with the aim of compressing morbidity in the later stages of the life course and promoting quality of life and well-being [1, 2]. The guidance is informed by evidence, which demonstrates the relationship between extent of social activity and morbidity and mortality in the extended lifespan [3] and the importance of participation in meaningful activities for mental well-being [4, 5].

An occupation-based intervention designed in the United States to promote continued participation and engagement (Lifestyle Redesign) was shown to be effective in improving the mental well-being of older adults through two randomised controlled trials [4, 6]. The aim of the study reported in this paper was to test whether an intervention modelled on Lifestyle Redesign and adapted for a UK population (Lifestyle Matters) could also demonstrate clinical and cost-effectiveness.

## Methods

### Study design

A pragmatic, multicentre randomised controlled trial was conducted in two contrasting UK sites (rural North Wales and a large urban city in Northern England) between December 2011 and November 2015. The study protocol was published [5]. A Trial Steering Group (TSC) and independent Data Monitoring Committee (DMC) were appointed to monitor the quality and conduct of the study.

### Participants

A variety of recruitment methods were used to attract community living people aged 65 years and over with reasonable cognitive ability to participate. The feasibility study had highlighted the value of using local communities to identify those who might benefit [7]. Therefore, considerable effort was invested in informing community health and social care practitioners and groups for older people through face to face meetings and media advertisements. However, the time constraints of undertaking a randomised controlled trial necessitated an additional recruitment strategy via general practicioner (GP) mail outs in the areas where intervention delivery was planned. The original intention was that mail outs would support achievement of recruitment targets within the required time frame [8].

### The intervention

Based on an occupational approach to healthy ageing, the manualised Lifestyle Matters intervention was designed to assist participants to improve their well-being and avoid the decline associated with social isolation and poor mental health (MH). Participants met in a weekly group of up to 12 people over 4 months at a local venue. Participants were also asked to engage in monthly individual sessions with a facilitator. Session topics were either chosen from the manualised programme or new topics identified [9]. The facilitators worked with the participants to explore the selected topic through discussion, activities and community enactment. The emphasis throughout was upon the identification of participants’ goals, empowerment through sharing strengths and skills and providing support to enable them to practice new or neglected activities independently, particularly in the community [10, 11]. The facilitators were paid National Health Service (NHS) or social care staff who were provided with training and supervised by qualified occupational therapists throughout.

### Study procedures

Eligible participants were enroled, screened for cognition and consented by a research assistant (RA) and randomised to one of two study arms (intervention or usual care) via a remote web-based randomisation service. The randomisation sequence was computer generated in advance by the trial statistician and stratified by site. Random permuted blocks of variable size were used to ensure that sufficient participants were allocated in a 50:50 ratio to each arm of the trial at each study site. When a couple in the same household both consented to take part, the pair was randomised as a couple.

The principal investigator (PI), TSC, study statisticians, health economists and RAs collecting outcome data at 6 and 24 months were blinded to treatment allocation but the Trial Manager, clerical team and participants were not blinded. RAs who undertook follow-up appointments asked participants to avoid revealing which arm they were allocated to.

All study participants were asked to participate in study data collection at baseline and follow-up.

Adherence to the manualised intervention was assessed [5, 9]. Facilitator fidelity to the group intervention was determined by two independent researchers evaluating video recordings of four groups (two at each site) during weeks 4 and 10 of delivery using a checklist which rated six domains: goals and needs, resources, personal qualities, enabling, group work skills and content. ‘Group member performance’ was also assessed using a checklist to determine a participant's uptake of the intervention and their understanding of it. Participant attendance at group and individual sessions was monitored through registers.

### Outcomes

All participants were assessed at baseline and followed up at 6 and 24 months post-randomisation using validated questionnaires, completed either face to face or over the telephone by an RA.

The primary outcome was mental well-being measured by the 36 Item Short Form Health Survey (SF-36) MH dimension score at 6 months [12], measured on a 0 (poor) to 100 (good health) scale. Secondary outcomes were other dimensions of the SF-36, Patient Health Questionnaire (PHQ) [13], EQ-5D-3 L [14], de Jong Gierveld Loneliness Scale [15], General Self-Efficacy Scale (GSE) [16] and Office for National Statistics (ONS) well-being at 6 and 24 months post-randomisation [17]. Serious adverse events (SAEs) were assessed at 6 and 24 months; these were assessed by the PI for relatedness to the intervention. Economic evaluation involved collection of all health and social care use over the previous 3 months at each data collection point through application of a bespoke health and social care resource use questionnaire.

### Statistical analysis

Sample size was derived from the mean SF-36 MH dimension score of a general health survey (68.3 with a standard deviation (SD) of 19.9) [18]. Assuming a mean difference in SF-36 MH scores of 8 or more is of a clinical or practical importance, and a SD of 20 points, to have an 80% power of detecting this difference, significant at the 5% (two-sided) level, with cluster sizes of 10 subjects per Lifestyle Matters group, an intra cluster correlation of 0.01 and with 20% lost to follow-up at 6 months, the study needed to recruit 268 participants (134 per arm). Primary and secondary outcomes were analysed using a linear mixed effects model with independent correlation and two levels of nested clustering. The lower level of clustering treats each couple as a cluster of size two, or each individual, if not in a couple, as a cluster of size one. The higher level of clustering regards participants in the same Lifestyle Matters intervention group as a cluster. Participants allocated to either arm who withdrew from intervention before being allocated a group were treated as a cluster of size one, or of size two if they were in a couple. An adjusted analysis was performed alongside this unadjusted analysis, which included potential baseline prognostic covariates of age, sex and baseline SF-36 MH dimension score and whether the participant lived alone in a mixed effects model. Analysis of secondary outcomes at 6 and 24 months post-randomisation was performed in a similar manner.

In calculating the cost-effectiveness of the intervention, a cost perspective of the NHS and social care was adopted. Intervention costs (cost of facilitators and their supervision, venue hire and related costs of delivering the intervention), drugs, inpatient stay, general practitioner visits, outpatient appointments, visits to the emergency department and day care services were included. Costs were obtained from NHS reference costs 2013–14 and other published sources [19, 20]. There was less than 5% missing data for costs and as a result no imputation was necessary. Costs and benefits had not been discounted. Utilities were calculated using SF-6D derived from SF-36 collected at baseline, 6 and 24 months. Quality-Adjusted Life Years (QALYs) were estimated using a total of 30 imputations and were calculated using the area under the curve method.

Cost-effectiveness was analysed using seemingly unrelated regression, a multivariate technique that takes into consideration potential correlation between costs and QALYs [21]. An incremental analysis was conducted by dividing mean incremental QALYs to produce an incremental cost-effectiveness ratio (ICER) by comparing participants in the intervention and control groups. Uncertainty in the decision is assessed from cost-effectiveness acceptability curve, which plots the probability that the intervention is cost-effective for a range of thresholds that the NHS would be willing to spend per QALY.

This work was supported by the Medical Research Council [grant number G1001406], who had no input into the design, execution, analysis and interpretation of data, or writing of the study.

## Results

**Figure 1.**
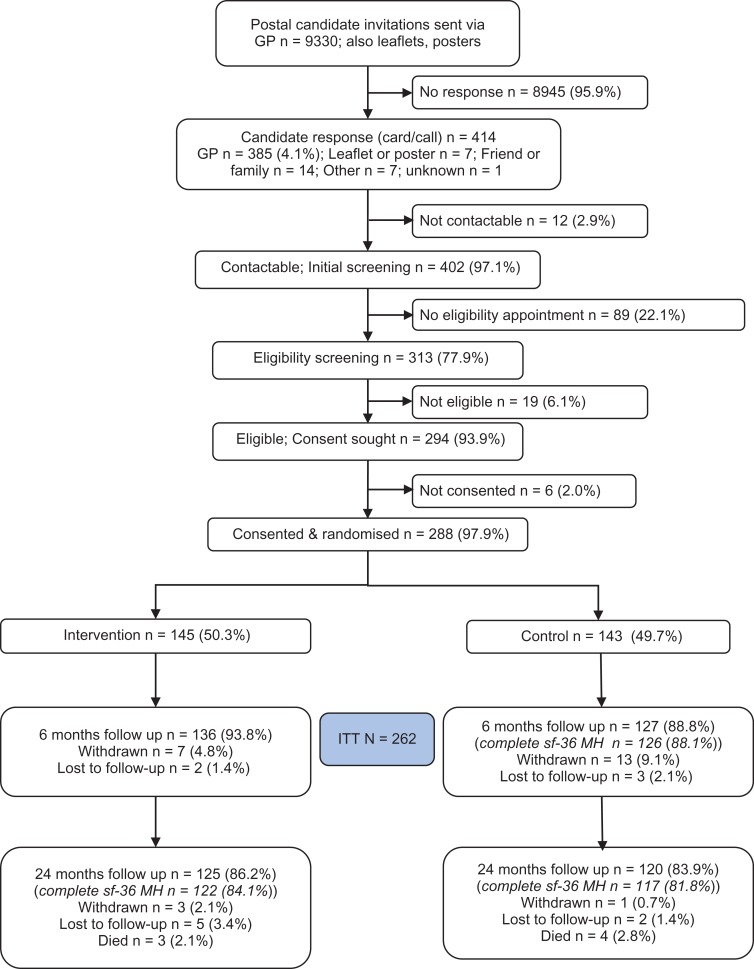
Trial profile.

The trial randomised 288 participants between 14 August 2012 and 19 April 2013 (18 couples and 252 individuals); 145 and 143 were allocated to the intervention and control groups, respectively (Figure 1). Twenty six participants either withdrew, were lost to follow-up, or had missing primary outcome data at 6 months, leaving 262 (91%) participants in the primary analysis (136 intervention; 126 control). Baseline characteristics of the participants are displayed in Table 1.

**Table 1.** Baseline characteristics by randomised group for participants in the ITT population

Characteristic	Intervention	Control	All
	(*n* = 145)	(*n* = 143)	(*n* = 288)
Sex, *n* (%)			
Male	44 (30.3%)	48 (33.6%)	92 (31.9%)
Female	101 (69.7%)	95 (66.4%)	196 (68.1%)
Age			
Mean (range)	72.9 (65-92)	71.3 (65-90)	72.1 (65-92)
Ethnic group, *n* (%)			
English/Welsh/Scottish/Northern Irish/British	142 (97.9%)	141 (98.6%)	283 (98.3%)
Irish	1 (0.7%)	1 (0.7%)	2 (0.7%)
European	1 (0.7%)	1 (0.7%)	2 (0.7%)
Prefer not to say	1 (0.7%)	0 (0.0%)	1 (0.3%)
Lives alone, *n* (%)	86 (59.3%)	71 (49.7%)	157 (54.5%)
Lives with, *n* (%)			
Spouse/partner	48 (33.1%)	61 (42.7%)	109 (37.8%)
Child/children	3 (2.1%)	4 (2.8%)	7 (2.4%)
Both partner and children	5 (3.4%)	6 (4.2%)	11 (3.8%)
Other	3 (2.1%)	1 (0.7%)	4 (1.4%)
Main activity/occupation, *n* (%)			
Employed or self employed	6 (4.1%)	6 (4.2%)	12 (4.2%)
Retired	133 (91.7%)	134 (93.7%)	267 (92.7%)
Looking after home/family	4 (2.8%)	2 (1.4%)	6 (2.1%)
Other	2 (1.4%)	1 (0.7%)	3 (1.0%)
If employed or retired; occupation type, *n* (%)			
Professional	27 (18.6%)	20 (14.0%)	47 (16.3%)
Managerial/technical	34 (23.4%)	33 (23.1%)	67 (23.3%)
Skilled (non-manual)	36 (24.8%)	39 (27.3%)	75 (26.0%)
Skilled (manual)	12 (8.3%)	24 (16.8%)	36 (12.5%)
Partly skilled	11 (7.6%)	10 (7.0%)	21 (7.3%)
Unskilled	18 (12.4%)	14 (9.8%)	32 (11.1%)
Age on leaving full time education			
*N* (%)	143 (98.6%)	141 (98.6%)	284 (98.6%)
Mean (SD)	16.4 (2.8)	16.2 (2.3)	16.3 (2.5)

A level, Advanced level; AS level, Advanced Subsidiary level; CSE, Certificate of Secondary Education; GCSE, General Certificate of Secondary Education; max., maximum; min., minimum; NVQ4, National Vocational Qualification level 4; O level, Ordinary level.

Intention to Treat (ITT) analysis found no difference in the primary outcome (SF-36 MH) between randomised groups (Table 2, adjusted mean difference 2.3, 95% CI: −1.3 to 5.9, *P* = 0.209). Fifty-two percent (71/136) of participants allocated to the intervention received a therapeutic dose of group sessions in that they attended 12 or more of the 16 weekly groups before 6-month follow-up. A sensitivity (per-protocol) analysis of the 71 participants who received a therapeutic dose of the group found similar results to the ITT analysis. Other sensitivity analyses, of the imputing missing data, gave consistent estimates of treatment difference (see Supplementary data, Table Appendix 1, available at *Age and ageing* online). There was no evidence of difference between those the intervention and usual care groups on any secondary outcomes at 6 months. However at 24 months, scores on two subscales (de Jong Gierveld Emotional Loneliness and de Jong Gierveld Social Loneliness) were significantly improved in the intervention compared to the usual care group (Table 2), although the relevance of this finding is questionable due to a lack of evidence to support a minimal clinically important difference [15]. Assessment of fidelity to the group component was satisfactory in seven out of eight video recordings for both group member and facilitator performance, indicating that the group component had been delivered as intended. The mean number of group sessions attended per participant was 9.2 (SD = 5.8). Out of the 123 participants who attended at least one group session, 93 (75.6%) were offered four individual sessions as per-protocol but only five (4.1%) accepted and received all four sessions. The number of participants that experienced a SAE was similar across the trial arms (46% (63/136) of the intervention and 53% (67/126) of the treatment as usual group). The majority of the SAEs reported persistent or significant disability/incapacity over the time scale since last assessment, which accounted for 71% of the total. All SAEs reported by intervention group participants were assessed as either unrelated or unlikely to be related to the intervention. RAs were unblinded to group allocation in 13.7% (*n* = 109) of follow-up appointments.

**Table 2.** ITT repeated measures analysis at baseline, 6 and 24 months post-randomisation.

Outcome	Baseline				6 months							24 months						
	Intervention		Control		Intervention		Control		Adjusted mean difference^a^	95% CI	*P*-value^b^	Intervention		Control		Adjusted mean difference^a^	95% CI	*P*-value^b^
	*n*	Mean (SD)	*n*	Mean (SD)	*n*	Mean (SD)	*n*	Mean (SD)				*n*	Mean (SD)	*n*	Mean (SD)			
SF-36 MH	145	75.5 (18.3)	143	77.0 (18.2)	136	77.3 (18.2)	126	75.9 (18.7)	2.3	−1.3 to 5.9	0.209	122	78.0 (17.1)	117	75.4 (17.8)	2.2	−1.4 to 5.8	0.233
SF-36 Physical function	145	67.5 (25.3)	143	71.7 (26.4)	136	66.0 (28.4)	126	70.7 (27.3)	1	−2.1 to 4.1	0.535	123	65.0 (27.8)	118	66.3 (29.5)	3	−0.7 to 6.8	0.116
SF-36 Role physical	145	72.4 (27.6)	143	76.8 (25.5)	136	69.9 (29.9)	126	73.9 (26.4)	0.9	−4.2 to 5.9	0.728	123	69.7 (27.5)	117	72.5 (27.7)	0.5	−5.1 to 6.1	0.855
SF-36 Bodily pain	145	61.2 (25.6)	143	64.7 (26.5)	136	60.5 (28.0)	126	61.6 (27.4)	1.9	−3.1 to 7.0	0.453	123	56.0 (25.6)	117	59.9 (26.1)	−1.1	−6.0 to 3.8	0.656
SF-36 General health	145	63.6 (20.4)	143	68.8 (20.4)	136	61.9 (22.7)	126	64.8 (21.1)	2.8	−0.6 to 6.2	0.103	123	64.3 (20.7)	117	64.0 (20.7)	3.4	−1.0 to 7.9	0.132
SF-36 Vitality	145	58.4 (21.4)	143	60.3 (20.9)	136	56.4 (22.2)	126	58.0 (21.7)	−0.2	−4.0 to 3.7	0.929	122	57.1 (21.6)	117	57.3 (19.5)	0.2	−3.7 to 4.2	0.902
SF-36 Social function	144	82.9 (22.0)	142	82.0 (26.4)	136	77.8 (28.2)	126	81.3 (26.0)	−3.7	−9.4 to 2.0	0.205	122	80.7 (25.4)	117	79.2 (25.2)	1.4	−4.3 to 7.1	0.63
SF-36 Role emotional	145	82.7 (23.4)	143	84.5 (21.5)	136	82.7 (23.2)	125	86.7 (19.4)	−2.4	−7.1 to 2.3	0.325	121	87.2 (20.2)	117	85.3 (22.9)	1.8	−3.1 to 6.7	0.466
SF-36 Physical component summary	144	44.1 (11.0)	142	45.9 (10.6)	136	42.8 (12.0)	125	44.4 (11.3)	1	−0.6 to 2.5	0.21	121	42.1 (11.5)	117	43.5 (11.6)	0.7	−1.2 to 2.5	0.487
SF-36 MCS	144	51.5 (10.4)	142	51.8 (10.0)	136	51.5 (9.7)	125	51.9 (10.1)	−0.3	−2.2 to 1.6	0.763	121	53.3 (9.9)	117	51.6 (10.0)	0.9	−1.1 to 2.9	0.384
EQ-5D-3L	142	0.73 (0.25)	143	0.77 (0.24)	133	0.71 (0.25)	126	0.76 (0.23)	−0.01	−0.05 to 0.03	0.742	121	0.73 (0.24)	116	0.71 (0.28)	0.05	−0.00 to 0.10	0.065
EQ-5D your health state today	145	73.0 (19.2)	142	77.7 (17.6)	135	72.6 (18.3)	126	77.3 (17.0)	−1.6	−5.0 to 1.9	0.37	121	74.7 (16.9)	118	75.3 (16.4)	0.9	−4.0 to 5.8	0.726
Brief Resilience Scale	143	3.6 (0.8)	140	3.6 (0.8)	132	3.7 (0.7)	123	3.7 (0.8)	0.1	−0.2 to 0.3	0.625	122	3.5 (0.8)	115	3.6 (0.8)	0	−0.2 to 0.2	0.872
de Jong Gierveld Emotional Loneliness	138	2.3 (2.1)	138	2.4 (2.0)	130	1.9 (2.0)	122	2.0 (2.1)	−0.2	−0.6 to 0.2	0.254	117	1.9 (2.1)	116	2.3 (2.2)	−0.5	−0.9 to −0.0	0.042
de Jong Gierveld Loneliness	142	4.1 (3.5)	142	4.6 (3.6)	134	3.5 (3.2)	124	4.1 (3.4)	−0.4	−0.9 to 0.2	0.201	121	3.7 (3.4)	117	4.8 (3.6)	−0.7	−1.4 to −0.1	0.026
de Jong Gierveld Social Loneliness	140	1.8 (1.8)	141	2.2 (1.9)	133	1.6 (1.8)	123	2.0 (1.9)	−0.1	−0.4 to 0.2	0.51	122	1.8 (1.8)	117	2.4 (1.9)	−0.2	−0.6 to 0.1	0.223
PHQ-9	143	4.1 (4.1)	135	3.3 (4.1)	133	3.8 (4.2)	122	3.4 (4.3)	−0.1	−0.9 to 0.6	0.762	122	3.8 (4.6)	114	4.0 (4.8)	−0.7	−1.6 to 0.2	0.122
GSE	144	31.7 (5.1)	143	31.9 (4.8)	135	31.9 (5.0)	124	31.6 (5.0)	0.5	−0.5 to 1.6	0.336	121	32.3 (5.1)	118	31.6 (5.4)	0.7	−0.4 to 1.9	0.213
ONS well-being	145	7.3 (2.2)	141	7.3 (2.2)	136	7.2 (2.4)	124	7.3 (2.3)	0	−0.4 to 0.4	0.911	120	7.4 (1.7)	115	7.3 (2.0)	0.1	−0.3 to 0.5	0.595

The Short Form (36) Health Instrument (SF-36) Dimensions are scored on a 0 (poor) to 100 (good) health scale, except for the Physical and MCS scores which are standardised to have a mean of 50 and SD of 10. The EuroQol 5-Dimension (EQ-5D) utility score is measured on a −0.56 to 1.00 (good health) scale. The EQ-5D visual analogue scale is measured on a 0 (worst imaginable health state) to 100 (best imaginable health state). The brief resilience scale is scored on a scale of 1–5 with higher scores indicating more resilience. The emotional loneliness scale of the De Jong is scored on a 0–6 scale with higher scores indicating more loneliness. The social loneliness scale of the De Jong is scored on a 0–5 scale with higher scores indicating more loneliness. The total loneliness scale of the De Jong is scored on a 0–11 scale with higher scores indicating more loneliness. The PHQ−9 is measured on a 0–27 scale with higher scores indicating more severe depressive symptoms. GSE Scale is scored on a 10–40 scale with higher scores indicating more perceived self-efficacy. The ONS instrument measures subjective well-being on a 0–40 scale, with higher scores indicating high subjective well-being. For the SF-36, EQ-5D, Brief Resilience Scale, GSE, ONS a positive mean difference indicates the invention group has the better QoL. For the de Jong Gierveld and PHQ-9 a negative mean difference indicates the Intervention group has the better QoL.

^a^Adjusted for lifestyle matters intervention group, couple, age, sex, baseline score and if lives alone for.

^b^*P*-value for adjusted mean difference between treatment and control conditions.

The cost of Lifestyle Matters was estimated at £430 and £575 (£1 = $1.51) per person in the North England and North Wales sites, respectively. From the regression analysis, the ICER was found to be £7,621 (see Supplementary data, Table Appendix 2, avaliable  at *Age and Ageing* online) but this lies in the third quadrant of the cost-effectiveness plane implying that the intervention is less costly but less effective. At a threshold of £20,000, commonly used within the NHS, there was a probability of 30% that Lifestyle Matters would be cost-effective. Utilities generated from EQ-5D to generate QALYs were used as sensitivity analysis. The incremental cost-effectiveness ratio was £7,861 but remained less costly and less effective.

## Discussion

This trial was undertaken to a high standard including blinding of outcome assessors, concealed randomisation techniques and recruitment to sample size. Follow-up was successful at 6 and 24 months post-randomisation (85% retention at 24 months). Limitations were that targeted recruitment through service providers and the community (recommended from the feasibility study) was unsuccessful, despite sustained effort [7]. To recruit the required numbers of participants meeting study eligibility criteria within the allocated time frame almost all were recruited through GP mail outs, resulting in a self-selecting sample.

The findings do not support the hypothesis that an intervention modelled on Lifestyle Redesign and adapted for a UK population (Lifestyle Matters) is effective at improving the well-being of older adults. The change in the primary outcome (MH dimension of the SF-36) over a 6-month period was not significantly different between the usual care and intervention groups [12]. Compared to the second US Lifestyle Redesign study, where recruited participants had a mean baseline SF-36 Mental component summary (MCS) score of 41, participants in our study were mentally well with mean baseline SF-36 MCS score scores of 52 [4] (MCS scores are standardised to have mean of 50 and SD of 10 the same as the reference population). Participants in the US studies were independently residing in retirement communities and private homes; those in private homes visited senior community centres. In our trial, older adults were also independently living but were recruited from the community and did not necessarily have any involvement in community centres. It can be deduced that participants recruited to Lifestyle Matters were not at a stage of their life when then would benefit most from such an intervention, nor were they activity seeking support when recruited. The US studies suggest that recruiting from an existing support group enabled recruitment of those with lowered mental well-being [4].

At 24 months there were significant decreases in aspects of emotional loneliness (e.g. ‘I often feel rejected’; ‘I miss having people around me’) for those who had participated in the Lifestyle Matters intervention. This suggests that the groups could have influenced a reappraisal of relationships and social networks, a potential area for further study. A small proportion of individuals (4.1%) took up all four offers of a one to one session with a facilitator. Fostering increased uptake of these sessions, which focussed on goal setting, may aid individuals gain quality of life in future evaluations.

Identifying older people at risk of mental decline and in particular those not known to services is challenging and has only recently been identified as a priority for UK health, social care and other agencies [2]. Consensus is required as to the responsibility of clinicians—especially GPs—for identifying such individuals, and the exact methods by which isolated older adults can be identified.

Identifying older people when they are beginning to decline and taking action at that point is crucial to the success of preventive interventions. Proactive recognition and signposting strategies are required, which were not in evidence during this study; the benefits of which were strongly indicated in our feasibility study [7]. Unlike the feasibility study, the randomised controlled trial methodology did not provide the time required to seek those in most need. We therefore do not know if those who are experiencing mental or physical decline would actually participate in and benefit from such an intervention. Identification of those in potential need remains an elusive challenge.

**Key points**

- Social participation and involvement in meaningful activities can prevent mental ill-health in older adults.
- Two US studies found that an occupation-based lifestyle intervention improved the mental well-being of older adults.
- We adapted the US lifestyle intervention for a UK population and assessed it's effectiveness in comparison to usual care.
- We were unable to recruit those with lowered mental well-being, which contributed to the intervention not showing effectiveness.
- Findings highlight the need for strategies to identify those who are on the cusp of decline.

## Supplementary Material

Supplementary Data
