# Supplementary material for: A preventative lifestyle intervention for older adults (lifestyle matters): a randomised controlled trial
Source: Age Ageing. 2017 Feb 25;46(4):627–34. doi: 10.1093/ageing/afx021 (PMC5860501; doi:10.1093/ageing/afx021)
Supplement: Supplementary Data [file supplementarymaterialageandageing.docx]

Supplementary material

Appendix 1

**Sensitivity analysis for primary outcome at 6 months**

|  | Treatment group | | | |  | |  | | | |  |  |  |  |  |
| --- | --- | --- | --- | --- | --- | --- | --- | --- | --- | --- | --- | --- | --- | --- | --- |
|  | Intervention | | Control | | | Unadjusted^a^ | | |  | Adjusted^b^ | | |  | |  |
| SF-36 MH  Outcome | n | Mean (SD) | n | Mean (SD) | | mean difference | | 95% CI | p-value | mean difference | | | 95% CI | | p-value |
|  |  |  |  |  | |  | |  |  |  | | |  | |  |
| Observed Data | 136 | 77.3 (18.2) | 126 | 75.9 (18.7) | | 1.3 | | -4.0 to 6.6 | 0.628 | 2.3 | | | -1.3 to 5.9 | | 0.209 |
|  |  |  |  |  | |  | |  |  |  | | |  | |  |
| Per-protocol | 71 | 80.3 (14.4) | 126 | 75.9 (18.7) | | 4.3 | | -3.4 to 12.0 | 0.278 | 3.1 | | | -0.9 to 7.2 | | 0.131 |
|  |  |  |  |  | |  | |  |  |  | | |  | |  |
| LOCF imputed data | 145 | 75.9 (19.5) | 143 | 75.0 (19.2) | | 0.4 | | -5.1 to 5.9 | 0.89 | 2 | | | -1.3 to 5.3 | | 0.241 |
|  |  |  |  |  | |  | |  |  |  | | |  | |  |
| Assuming 10 point decrease | 145 | 75.3 (20.3) | 143 | 73.8 (19.9) | | 0.5 | | -5.3 to 6.3 | 0.864 | 2.6 | | | -0.9 to 6.0 | | 0.146 |
|  |  |  |  |  | |  | |  |  |  | | |  | |  |
| Multiple imputation^c^ | 145 | 76.3 (18.8) | 143 | 74.9 (19.3) | | 0.9 | | -4.6 to 6.4 | 0.74 | 2.4 | | | -1.1 to 5.9 | | 0.186 |

The SF-36 mental health dimension is scored on a scale from 0 (poor) to 100 (good). a. Adjusted for lifestyle matters intervention group and couple. b. Adjusted for lifestyle matters intervention group, couple, age, sex, baseline SF-36 MH score, and if lives alone. c. Multiple imputation using chained equations (regression) based on 20 imputed data sets, with age, sex and baseline score, lives alone, baseline EQ-5D your health state today and baseline de Jong Gierveld overall loneliness as covariates. All analyses use a mixed effects regression model, with nested clustering of couples/individuals within lifestyle matters intervention groups.

Appendix 2

**Cost-utility results**

|  | **Using SF-6D to calculate QALYs** | | | **Using EQ-5D to calculate QALYs** | | |
| --- | --- | --- | --- | --- | --- | --- |
|  | Mean | SD | CI (95%) | Mean | (SD) |  |
| Incremental costs £ | - 222 | 570 | -1338 to 894 | -222 | 570 | -1338 to 894 |
| Incremental QALYs | -0.029 | 0.016 | -0.060 to 0.002 | -0.028 | 0.028 | -0.08 to 0.026 |
| ICER | **£7621** | | | **£ 7861** | | |
